# Enhanced Functional Coupling of Hippocampal Sub-regions in Congenitally and Late Blind Subjects

**DOI:** 10.3389/fnins.2016.00612

**Published:** 2017-01-10

**Authors:** Guangyang Ma, Dan Yang, Wen Qin, Yong Liu, Tianzi Jiang, Chunshui Yu

**Affiliations:** ^1^Department of Radiology and Tianjin Key Laboratory of Functional Imaging, Tianjin Medical University General HospitalTianjin, China; ^2^Key Laboratory of Hormones and Development (Ministry of Health), Tianjin Key Laboratory of Metabolic Diseases, Tianjin Metabolic Diseases Hospital & Tianjin Institute of Endocrinology, Tianjin Medical UniversityTianjin, China; ^3^Tianjin Central Hospital of Gynecology ObstetricsTianjin, China; ^4^Brainnetome Center, Institute of Automation, Chinese Academy of SciencesBeijing, China

**Keywords:** functional connectivity, hippocampus, congenitally blind, late blind, resting state

## Abstract

The hippocampus has exhibited navigation-related changes in volume and activity after visual deprivation; however, the resting-state functional connectivity (rsFC) changes of the hippocampus in the blind remains unknown. In this study, we focused on sub-region-specific rsFC changes of the hippocampus and their association with the onset age of blindness. The rsFC patterns of the hippocampal sub-regions (head, body and tail) were compared among 20 congenitally blind (CB), 42 late blind (LB), and 50 sighted controls (SC). Compared with the SC, both the CB and the LB showed increased hippocampal rsFCs with the posterior cingulate cortex, angular gyrus, parieto-occpital sulcus, middle occipito-temporal conjunction, inferior temporal gyrus, orbital frontal cortex, and middle frontal gyrus. In the blind subjects, the hippocampal tail had more extensive rsFC changes than the anterior hippocampus (body and head). The CB and the LB had similar changes in hippocampal rsFC. These altered rsFCs of the hippocampal sub-regions were neither correlated with onset age in the LB nor the duration of blindness in CB or LB subjects. The increased coupling of the hippocampal intrinsic functional network may reflect enhanced loading of the hippocampal-related networks for non-visual memory processing. Furthermore, the similar changes of hippocampal rsFCs between the CB and the LB suggests an experience-dependent rather than a developmental-dependent plasticity of the hippocampal intrinsic functional network.

## Introduction

Sighted people mainly depend on the visual system to locate objects and to navigate through their environment. It is interesting to know how blind people use limited spatial information conveyed by the remaining sensory modalities to accomplish these functions. Blind subjects usually exhibit comparable or even superior non-visual spatial abilities compared to sighted subjects, including sound localization (Lessard et al., 1998; Gougoux et al., 2005), tactile orientation (Van Boven et al., 2000; Norman and Bartholomew, 2011), and navigation (Fortin et al., 2008; Kupers et al., 2010), although some studies report an impaired performance (Zwiers et al., 2001; Gori et al., 2014). During the non-visual spatial-related tasks, the occipital cortex has frequently been found to be activated in the early blind (EB) (Arnott et al., 2013), such as middle temporal area (MT) activation in motion discrimination and dorsal visual pathway activation in spatial discrimination and navigation (Cohen et al., 1997; Weeks et al., 2000; Vanlierde et al., 2003; Gougoux et al., 2005; Poirier et al., 2006; Ricciardi et al., 2006; Voss et al., 2006; Kupers et al., 2010; Matteau et al., 2010; Gagnon et al., 2012). The cross-modal activation of the occipital cortex has been associated with the superior non-visual spatial abilities in the EB (Gougoux et al., 2005; Simon-Dack et al., 2008). Furthermore, early studies had reported newly established anatomical connectivities (Bridge et al., 2008) between the subcortical visual relays and the intact visual areas, and reshaped effective connectivities (Silvanto et al., 2007, 2009) in both the ipslesional and contralesional spared visual areas after cortical blindness. However, the reorganization of the hippocampus, a structure for spatial mapping and navigation, in the blind is seldom considered.

The involvement of the hippocampus in processing spatial information is evidenced by both hippocampal activation during navigation tasks (Iaria et al., 2003) and spatial memory impairment due to hippocampal lesions (Bohbot et al., 1998). The processing precision of spatial memory in the hippocampal formation (HF) varies along its longitudinal axis. For example, the fields (a measure of neuronal spatial scale) of place cells are smaller in the posterior HF than in the anterior HF in rats, indicating that the posterior hippocampus processes spatial memory with more details (Jung et al., 1994; Maurer et al., 2005; Kjelstrup et al., 2008). Moreover, the anterior hippocampus is connected with the anterior temporal system that mainly supports object and verbal memory, whereas the posterior hippocampus is connected with the posterior medial system that mainly supports memory for scene and spatial layout (Ranganath and Ritchey, 2012), indicating segregated memory functions of the HF along its longitudinal axis.

The size of the hippocampus depends on experience. A species with a larger hippocampal volume indicates that spatial memory is more important to its survival (Barnea and Nottebohm, 1994; Clayton and Krebs, 1994; Lee et al., 1998). London taxi drivers show increased volume in the posterior hippocampus with an increase in years of experience, suggesting that the structural organization of the hippocampus is shaped by navigational experience (Maguire et al., 2000). The spatial navigation of blind subjects is challenged by their lack of visual input because only limited spatial information can be supplied by non-visual sensory modalities. Thus, the hippocampus of blind subjects may reorganize itself to adapt to these changes. In this context, several studies have reported reduced volume in the posterior area and increased volume in the anterior area of the right hippocampus in blind people (Chebat et al., 2007; Fortin et al., 2008; Leporé et al., 2009). Moreover, congenitally blind (CB) but not blindfolded sighted subjects have shown increased activation in the middle and posterior areas of the right hippocampus when they perform a tactile T-maze navigation task (Gagnon et al., 2012). These findings confirm experience-dependent plasticity in the hippocampus in the blind, and suggest a sub-region-dependent reorganization. However, the reorganization of intrinsic functional network of the hippocampal sub-regions in the blind remains unknown. Recent studies showed that spatial navigation needs the synergism of distributed brain areas which constitute a navigational-related network, including hippocampus, parahippocampal cortex (PHC), posterior cingulate cortex (PCC), retrosplenial cortex (RSC), dorsal occipital-parietal pathway, inferior temporal gyrus (ITG), prefrontal cortex (PFC), orbital frontal cortex (OFC), angular gyrus (AG), and anterior thalamus (Committeri et al., 2004; Feierstein et al., 2006; Kupers et al., 2010; Kravitz et al., 2011). Among these brain regions, the hippocampus is a critical hub of the navigational-related network. Measuring the potential connectivity changes of the hippocampus may shed light on navigational-related functional reorganization after visual deprivation.

In blind people, experience-dependent plasticity may interact with the developmental stages at which the individuals lost their sight. In this context, EB (or CB) and late blind (LB) subjects have exhibited different changes patterns in cortical thickness (Jiang et al., 2009; Park et al., 2009; Kupers et al., 2011), glucose metabolism (Wanet-Defalque et al., 1988; Veraart et al., 1990), task-evoked activation (Büchel et al., 1998), and functional connectivity density (FCD) (Qin et al., 2015) in the occipital cortex. However, the influence of the onset age of blindness on the intrinsic functional reorganization of the hippocampus remains unclear.

In this study, we compared group differences in resting-state functional connectivity (rsFC) of the hippocampal sub-regions among CB (*n* = 20), LB (*n* = 42) and sighted control (SC, *n* = 50) participants. Because the anterior and posterior hippocampal regions demonstrate different structural changes in the blind (Chebat et al., 2007; Fortin et al., 2008; Leporé et al., 2009) and they demonstrate different contributions on spatial processing in sighted subjects (Ranganath and Ritchey, 2012), we segmented the hippocampus into head, body and tail along the anterior-posterior axis. We hypothesize that the hippocampal sub-regions may exhibit different rsFC changes after visual deprivation. Based on the interaction between experience-dependent plasticity of the brain and the onset age of blindness (Voss, 2013), we also hypothesize that the rsFC changes of the hippocampus may be different between the CB and the LB.

## Materials and methods

### Subjects

This study included 20 CB (13 males; mean age: 26.6 ± 5.0 years, age range: 20–39 years), 42 LB (onset age of blindness >12 years; 30 males; mean age: 30.2 ± 5.8 years, age range: 21–45 years), and 50 SC (33 males; mean age: 28.8 ± 7.0 years, age range: 19–44 years). All the subjects were peripheral blindness, right handed, and they had no history of neurological or psychiatric disorders. In addition, all of the blind subjects had no pattern vision. Demographic data of these subjects are shown in Table 1 and detailed demographic information are shown in Table S1. The mean onset age of blindness in the LB was 19.1 ± 5.0 years. The mean duration of blindness was 26.6 ± 5.0 and 11.1 ± 5.3 years for the CB and the LB, respectively. There were no significant differences in age (one-way ANOVA, *F* = 2.34, *P* = 0.10) and gender (chi-square test, χ^2^ = 0.14, *P* = 0.93) among the three groups. This experiment was approved by the Medical Research Ethics Committee of Tianjin Medical University, and all participants gave their written informed consent.

**Table 1:** **Demographic data of the subjects**.

**Groups**	**Gender**	**Age (years)**	**Age of blindness onset (years)**	**Duration of blindness (years)**
CB (M/F)	13/7	26.60 ± 5.02	0	26.60 ± 5.02
LB (M/F)	29/13	30.24 ± 5.78	19.14 ± 5.40	11.10 ± 5.29
SC (M/F)	33/17	28.76 ± 6.99		
Statistics	χ^2^ = 0.14	*F* = 2.36		
*P* values	0.93	0.10		

*Data are presented as mean ± standard deviation. CB, congenitally blind; F, female; LB, late blind; M, male; SC, sighted controls*.

### MRI acquisition

The MR images were obtained using a 3.0-Tesla MR scanner (Trio Tim system; Siemens, Erlangen, Germany) that was equipped with a 12-channel head coil. Tight but comfortable foam padding was used to minimize head motion, and earplugs were used to reduce scanner noise. The acquisition of structural images used a 3D magnetization-prepared rapid-acquisition gradient echo (MPRAGE) sequence with the following parameters: repetition time (TR)/echo time (TE)/inversion time (TI) = 2000/2.6/900 ms, flip angle = 9°, matrix = 256 × 224, field of view (FOV) = 256 mm × 224 mm, slice thickness = 1 mm, and 176 continuous sagittal slices. The acquisition of resting-state fMRI data used a single-shot gradient-echo echo-planar imaging (SS-GRE-EPI) sequence: TR/TE = 2000/30 ms, matrix = 64 × 64, flip angle = 90°, FOV = 220 × 220 mm, 32 interleaved axial slices, thickness = 3 mm, slice gap = 1 mm, time points = 180. During the fMRI scans, all subjects were instructed to keep their eyes closed, relax, move as little as possible, think of nothing in particular and remain awake. After the fMRI scan, the fMRI images and the subjects' conditions were checked to confirm whether they satisfied the requirement, and if they did not, the fMRI data were abandoned and the subjects were rescanned.

### Data preprocessing

The resting-state fMRI data were preprocessed using Statistical Parametric Mapping (SPM8; http://www.fil.ion.ucl.ac.uk/spm). The first 10 volumes were discarded to remove signal shift caused by incomplete T1-relaxation. The remaining 170 volumes were then corrected for timing difference between slices. Rigid realignment was used to correct the displacement between volumes. During this process, the three translational and three rotational motion parameters as well as frame-wise displacement (FD) were calculated (Power et al., 2012). If the fMRI data had a maximum translational displacement higher than 1 mm or a maximum rotational displacement higher than 1.0°, the dataset of this subject would be discarded. All subjects' fMRI data were within defined motion thresholds. There were no significant differences in FD (one-way ANOVA, *F* = 0.33, *P* = 0.72) among the three groups, indicating that head motion might not contaminate the inter-group comparisons in rsFC. The 6 rigid motion parameters, and their first-order derivation, the mean signal of cerebrospinal fluid and white matter, and the spike point (time point with FD higher than 0.5) were regressed from data. The datasets were then band-pass filtered with a frequency range of 0.01–0.08 Hz. Individual structural images were linearly coregistered to the mean functional image; then the transformed structural images were segmented into gray matter (GM), white matter, and cerebrospinal fluid. The GM maps were linearly coregistered to the tissue probability maps in the Montreal Neurological Institute (MNI) space. Finally the motion-corrected functional volumes were spatially normalized to the MNI space using the parameters estimated during linear coregistration. The functional images were resampled into 3 × 3 × 3 mm^3^ voxels. After normalization, all datasets were smoothed with a Gaussian kernel of 6 × 6 × 6 mm^3^ full-width at half maximum (FWHM).

### Extraction of the hippocampal sub-regions and rsFC computation

**Figure 1:**
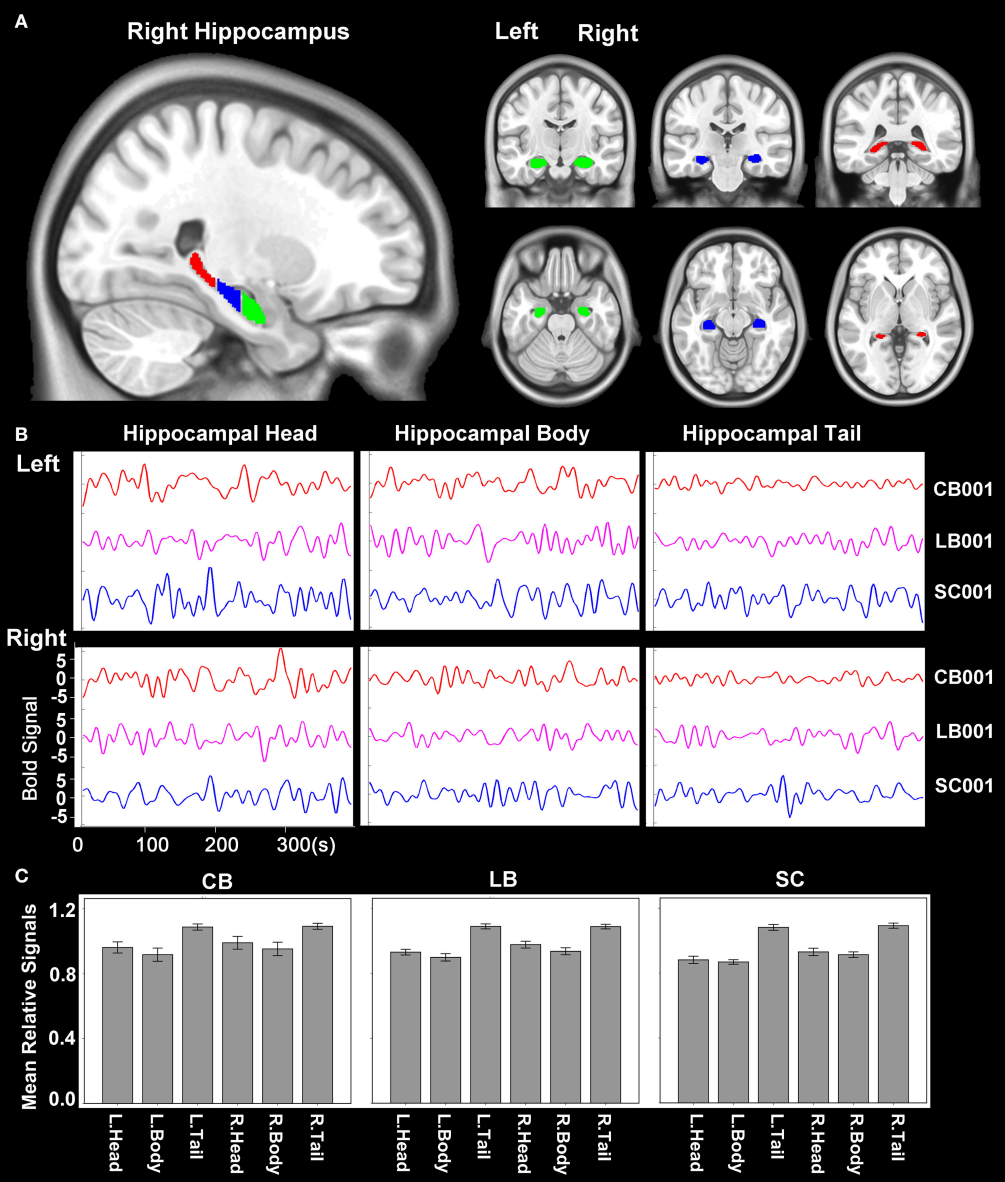
**Hippocampal sub-regions. (A)** The hippocampus is segmented into 3 equal-long areas along the longitudinal axis, which are shown in the sagittal, coronal and axial planes, respectively. The green, blue and red areas represent the hippocampal head, body and tail, respectively. **(B)** An example of the time course of BOLD signal of each hippocampal sub-region of one subject from each group. **(C)** The mean relative signal of each hippocampal sub-region of each group. The relative signal of a sub-region is calculated as the quotient between the average signal of each sub-region and that of the whole brain of each subject. BOLD, blood oxygen level dependent.

The hippocampus was segmented using the probabilistic map of Harvard-Oxford cortical and subcortical structural atlases (implemented in the FSL package) with a probabilistic threshold higher than 50%. Each side of the hippocampus was then trisected into head, body and tail along the anterior-posterior axis of the MNI space (Figure 1A). Then the time courses of average blood oxygen level dependent (BOLD) signals of the six sub-regions of the hippocampus (head, body and tail in each side) were extracted for the calculation of rsFC (Figure 1B). For an individual dataset, Pearson's correlation coefficients between the mean time series of each defined regions of interest (ROIs) and the time series of each voxel in other parts of the brain GM were computed and converted to *z* values using Fisher's *r*-to-*z* transformation to improve the normality. We also calculated the relative signal intensity of each hippocampal sub-region (relative to the whole brain) for each subject. The mean relative signal intensity of each hippocampal sub-region of each group is shown in Figure 1C. The mean relative signal intensities of hippocampal sub-regions were acceptable and comparable across groups (ranges: 0.72–1.17 for the CB, 0.68–1.23 for the LB, and 0.71–1.27 for the SC), suggesting that the rsFC changes of hippocampal sub-regions cannot be simply explained by artifact or abnormality of the BOLD signals.

### Statistical analysis

One-sample *t*-tests were used to identify brain regions showing positive rsFC with each hippocampal sub-region of each group within the brain gray matter mask. A Monte Carlo simulation method was used to correct for multiple comparisons with a corrected threshold of *P* < 0.05 (AlphaSim program, parameters including: single voxel *P* = 0.01, 5000 simulations, estimated FWHM, cluster connection radius = 5 mm) (Song et al., 2011). Then, a one-way analysis of covariance (ANCOVA) was used to test the rsFC differences of the hippocampal sub-regions among the CB, LB and SC controlling for age and gender effects (*P* < 0.05, AlphaSim corrected). For each hippocampal sub-region, the ANCOVA analysis was restricted to a mask that showed positive rsFC in at least one group. This mask was generated by adding the significant maps of one-sample *t*-tests of the three groups. For each subject, the ROIs that showed significant group differences in rsFCs were extracted for *post-hoc* analyses using a general linear model with the group as the independent variable and gender and age as nuisance covariates (*P* < 0.05). Partial correlation coefficients between the rsFC and the onset age of blindness in the LB, and those between the rsFC and the duration of blindness in the CB and LB, were also analyzed when controlling for the gender effect (*P* < 0.05).

## Results

### The rsFC patterns of the hippocampal sub-regions

**Figure 2:**
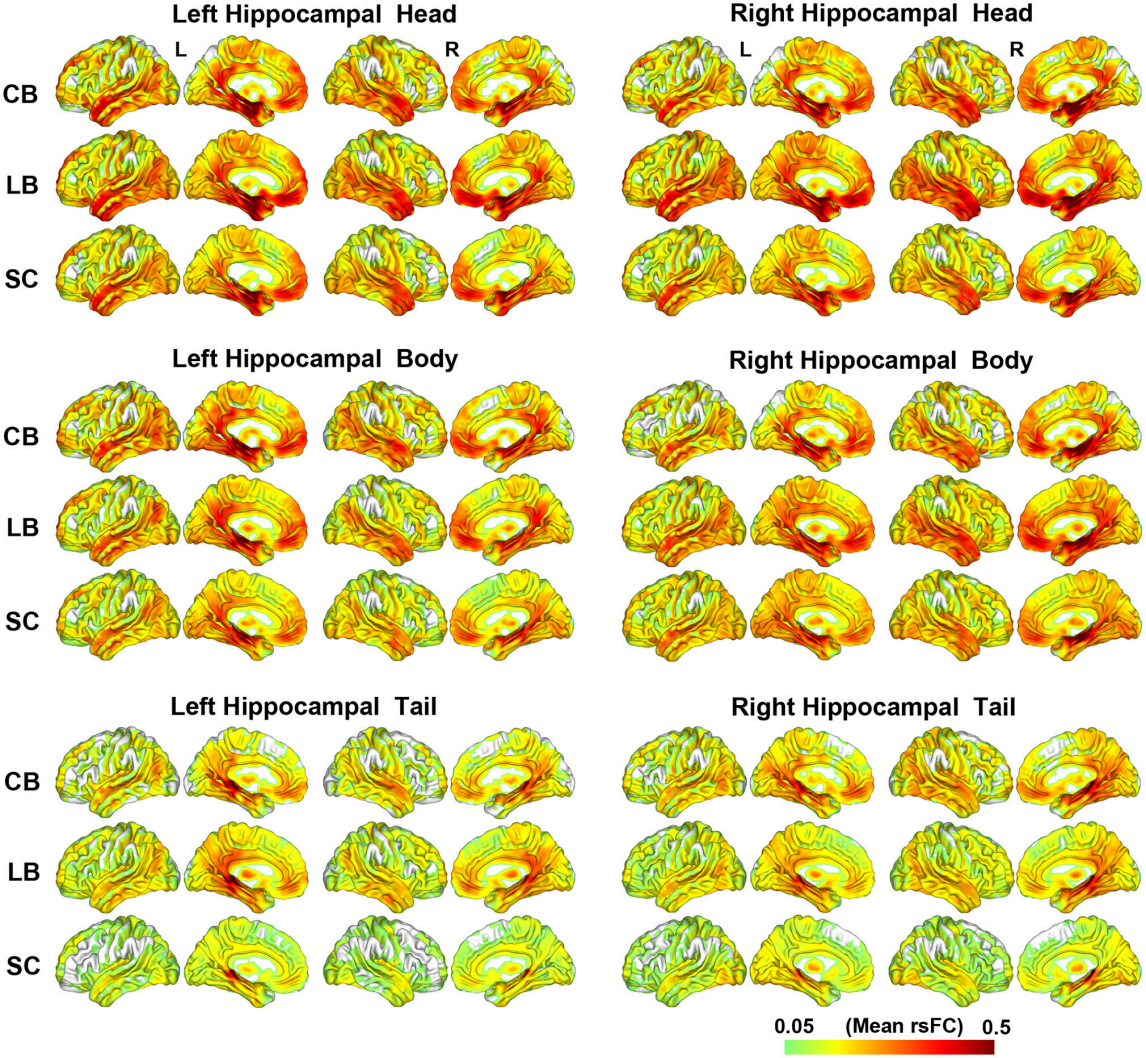
**The resting-state functional connectivity patterns of the hippocampal sub-regions**. CB, congenitally blind; LB, late blind; rsFC, resting-state functional connectivity; SC, sighted controls.

The rsFC patterns of each hippocampal sub-region of each group are shown in Figure 2. Although, the hippocampal tails had relatively weaker rsFCs than the hippocampal heads and bodies, these three hippocampal sub-regions were all strongly connected with the medial (MTL) and lateral temporal lobes (LTL), thalamus, PCC and middle cingulate cortex (MCC), medial prefrontal cortex (MPFC), and occipito-temporal conjunction.

### The changes in rsFC of the hippocampal sub-regions in the blind

**Figure 3:**
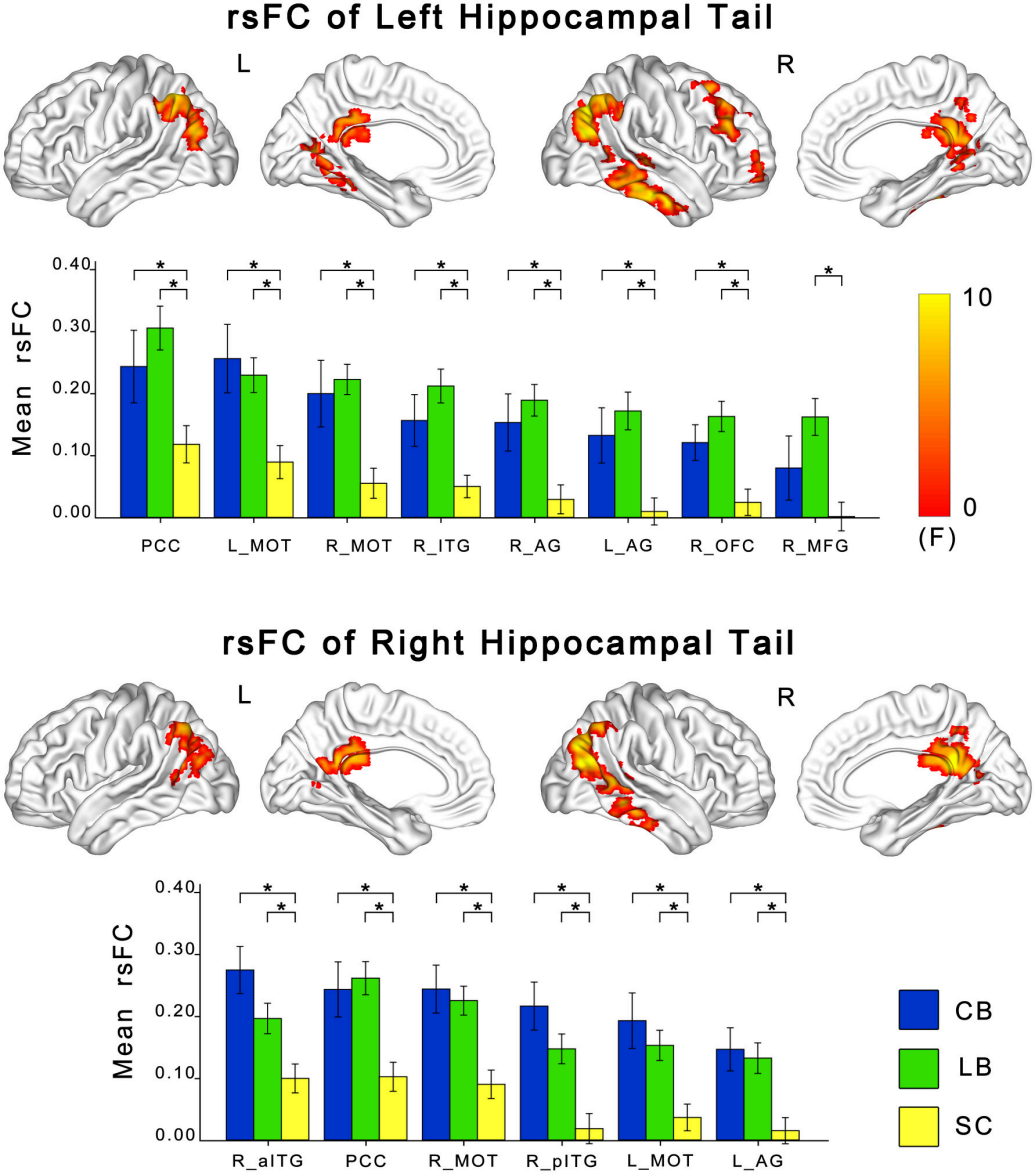
**Differences in resting-state functional connectivity of hippocampal tails among the CB, the LB and the SC**. For each sub-region, the upper panel shows brain regions exhibiting significant intergroup differences in rsFC with the hippocampal head or body (*P* < 0.05, corrected), and the lower panel shows *post-hoc* comparisons. Asterisk denotes significant rsFC difference between groups (*P* < 0.05, uncorrected). Error bars indicate the standard error of the mean. AG, angular gyrus; CB, congenitally blind; ITG, inferior temporal gyrus; LB, late blind; MFG, middle frontal gyrus; MOT, middle occipital temporal conjunction; OFC, orbital frontal cortex; PCC, post cingulate cortex; rsFC, resting-state functional connectivity; SC, sighted controls.

**Figure 4:**
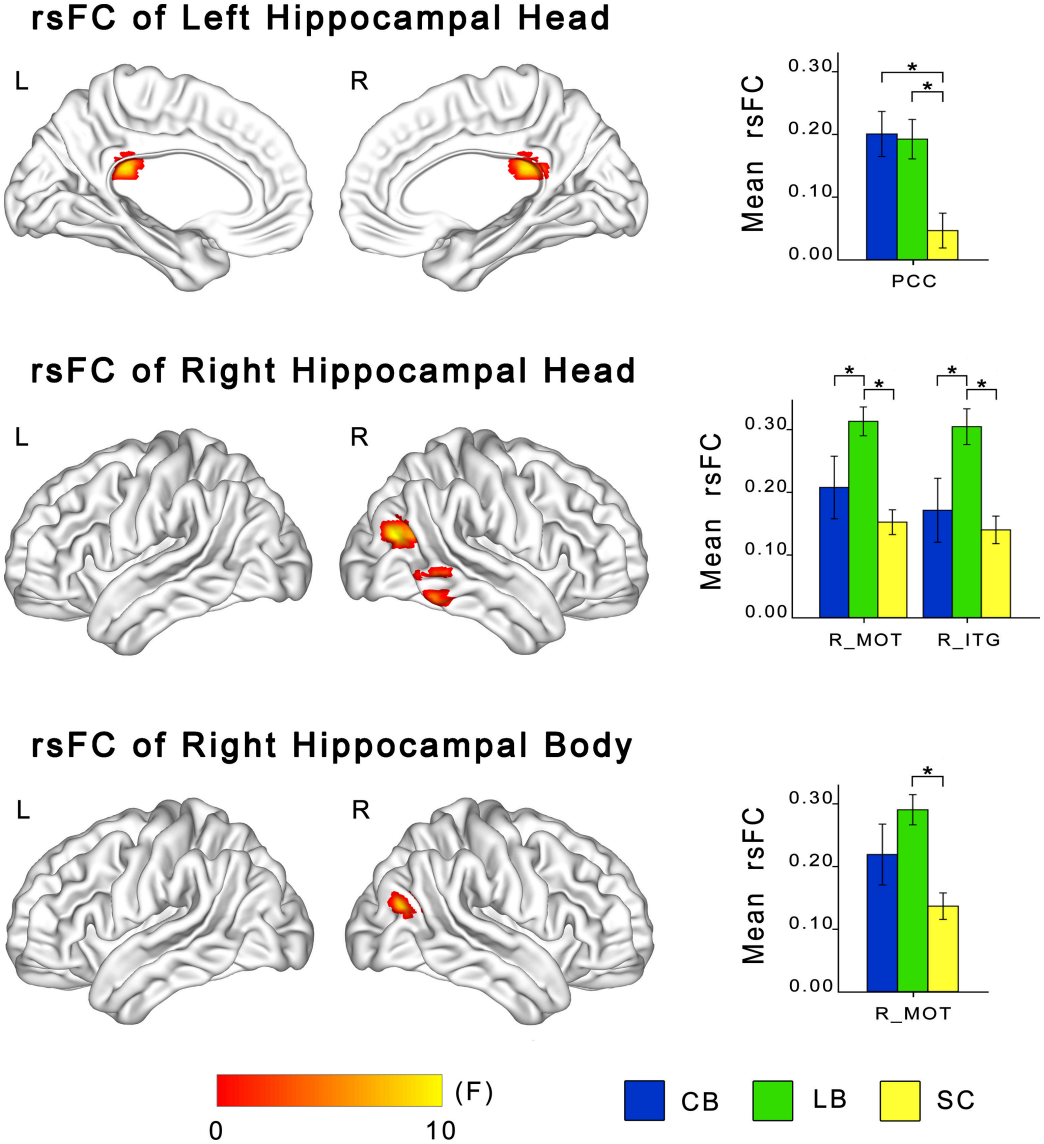
**Differences in resting-state functional connectivity of hippocampal heads and bodies among the CB, the LB and the SC**. For each sub-region, the left panel shows brain regions exhibiting significant intergroup differences in rsFC with the hippocampal sub-region (*P* < 0.05, corrected), and the right panel shows *post-hoc* comparisons. Asterisk denotes significant rsFC difference between groups (*P* < 0.05, uncorrected). Error bars indicate the standard error of the mean. CB, congenitally blind; ITG, inferior temporal gyrus; LB, late blind; MOT, middle occipital temporal conjunction; PCC, post cingulate cortex; rsFC, resting-state functional connectivity; SC, sighted controls.

ANCOVA showed significant differences in rsFCs of the bilateral hippocampal sub-regions among the CB, the LB, and the SC (Figures 3, 4). Altered rsFCs were present between the left hippocampal tail and the bilateral AG, PCC, parieto-occpital sulcus (POS), bilateral middle occipito-temporal conjunction (MOT), right ITG, right lateral OFC and right middle frontal gyrus (MFG), between the right hippocampal tail and the left AG, PCC, bilateral MOT, and right ITG, between the left hippocampal head and the PCC, between the right hippocampal body and the right MOT, and between the right hippocampal head and the right MOT and right ITG. There was no significant alteration in the rsFC of the left hippocampal body. In general, the rsFCs of the hippocampal tails showed more extensive changes among the three groups than those of the bodies and heads.

The *post-hoc* analyses revealed that both blind groups showed significantly strengthened rsFCs with the hippocampus compared to the SC group (Figures 2, 3). Specifically, the rsFC between the left hippocampal tail and the right MFC and between the right hippocampal body and the right MOT were increased in only the LB. Moreover, the LB exhibited increased rsFC between the right hippocampal head and the right OFC and ITG compared to the CB and the SC. The remaining brain regions had strengthened rsFCs with the hippocampal sub-regions in both the CB and the LB.

### Correlations between the hippocampal rsFC and the onset age and duration of blindness in the blind subjects

Partial correlation analyses showed that the onset age of blindness was not correlated with the rsFCs of any hippocampal sub-regions in LB subjects. The duration of blindness was not correlated with the rsFCs of any hippocampal sub-regions in either the CB or LB subjects (Table S2, Figures S1–S3).

## Discussion

In this study, we investigated the alterations in rsFCs of the hippocampal sub-regions after visual deprivation and found that these sub-regions (especially the hippocampal tails) had increased functional connectivity with several navigational-related areas. Furthermore, we found the alteration patterns of hippocampal functional connectivity were dramatically similar between the CB and the LB.

### Strengthened connectivity in the hippocampal-related functional network in the blind

We detected enhanced rsFCs between the hippocampus and the MOT, PCC, RSC, AG, ITG, MFG, and OFC, most of which are components of navigational-related areas (Committeri et al., 2004; Feierstein et al., 2006; Kupers et al., 2010; Kravitz et al., 2011). The MOT is an important component of the occipital-parietal circuit, which integrates information from the visual fields and represents space mainly in an egocentric frame of reference (Kravitz et al., 2011). Our finding of increased rsFC between the hippocampus and the MOT in the blind was consistent with previous studies showing cross-modal activation of the middle occipital cortex and MT+ for spatial processing in the CB/EB (Weeks et al., 2000; Burton et al., 2003; Poirier et al., 2006; Saenz et al., 2008; Arnott et al., 2013; Collignon et al., 2013) and the LB (Collignon et al., 2013), and was also consistent with structural and activation changes in the hippocampus after visual deprivation (Chebat et al., 2007; Fortin et al., 2008; Leporé et al., 2009; Gagnon et al., 2012). Our findings provided a potential link between the occipital and hippocampal reorganization after visual deprivation.

The PCC and RSC are important hubs in the parieto–medial temporal pathway that mainly supports spatial navigation (Kravitz et al., 2011). The PCC/RSC and AG have reciprocal connections with the hippocampus, PHC, anterior thalamic nuclei, and mammillary bodies (Taube, 2007; Buckwalter et al., 2008; Boccara et al., 2010; Uddin et al., 2010), which constitute a network for spatial memory and navigation. The PCC/RSC are closely related to spatial memory and navigation and involved in coordinating and translating between egocentric and allocentric reference frames (Knight and Hayman, 2014). As a result, the increased rsFCs between the hippocampus and these spatial processing hubs (PCC, RSC, and AG) in our study may represent functional reorganization within the navigation network after visual deprivation.

The MFG is the highest terminal of the parieto–prefrontal pathway (Schall et al., 1995). It has direct connections with the IPL and MT+ (Sakata and Kusunoki, 1992; Clower et al., 2005). The MFG is involved in top-down control of visual-spatial processing, such as spatial attention and working memory (Friedman and Goldman-Rakic, 1994). The increased rsFC between the hippocampus and the MFG may indicate the increased integration between the cognitive control network and the hippocampal-related network in the blind.

The ITG also had strengthened rsFC with the hippocampus in the blind. As a component of the ventral visual stream, the ITG is connected with parahippocampal cortex (Webster et al., 1991), which is a necessary relay for hippocampal signal transmitting (Insausti et al., 1987). Thus, our finding of increased rsFC between the hippocampus and the ITG may represent functional reorganization of the ventral visual stream. Actually, the CB/EB subjects have shown cross-modal plasticity in many areas of the ventral visual stream, such as the parahippocampal place area (PPA) for objects processing (He et al., 2013), the visual word form area (VWFA) for Braille reading (Reich et al., 2011), and the ITG for auditory shape (Striem-Amit et al., 2012) and Braille semantic processing (Melzer et al., 2001; Burton et al., 2003). Furthermore, the ITG is involved in navigation tasks in both sighted and congenitally blind subjects (Kupers et al., 2010), indicating a general role of this area in navigation processing.

The OFC also showed increased rsFC with the hippocampus. The OFC directly connects to brain regions for spatial processing, including the posterior parietal cortex, hippocampus, PHC, PCC, and RSC (Cavada et al., 2000). Although, the OFC is frequently reported in reward processing (Kahnt et al., 2010; Riceberg and Shapiro, 2012), it also participants in spatial navigation (Vafaei and Rashidy-Pour, 2004; Feierstein et al., 2006). The increased rsFC between the hippocampus and the OFC is also consistent with a recent study reporting increased FCD in these two regions in both the CB and LB (Qin et al., 2015).

### More extensive increase in rsFC of the hippocampal tails than the anterior parts

We found that the hippocampal tails showed more extensive increase in rsFC than the hippocampal heads and bodies in the blind, which is consistent with a previous study that showed increased activation in the posterior and middle areas of the hippocampus when CB subjects performed a tactile T-maze navigation task (Gagnon et al., 2012). However, these functional enhancements seem contrary to the observation that the volume in the posterior hippocampus was reduced in the blind (Chebat et al., 2007), and contrary to the report of no changed hippocampal activation during virtual route recognition in the CB (Kupers et al., 2010). The posterior hippocampus prefers to process memory for scene and spatial layout (Köhler et al., 2002), and structural plasticity of the posterior hippocampus has been associated with navigation experience in London taxi drivers (Maguire et al., 2000). In this context, the enhanced rsFCs of the hippocampal tails may explain the compensatory behavior for non-visual spatial navigation in the blind (Fortin et al., 2008; Kupers et al., 2010). In sighted people, visual information is critical for the hippocampus to construct spatial memory; visual deprivation may directly induce the disuse atrophy of the neurons and connections that normally serve visual processing in the posterior hippocampus, which may explain the decreased volume in the posterior hippocampus in the blind. However, the neurons and connections of the posterior hippocampus that serve non-visual processing may be strengthened due to experience-dependent plasticity in the blind. This hypothesis may explain why the blind subjects have strengthened rsFC (of our finding) and activation (Gagnon et al., 2012) of the posterior hippocampus and exhibit comparable or even superior non-visual spatial navigation abilities (Fortin et al., 2008; Kupers et al., 2010). It should be noted that the hippocampal activation by non-visual spatial navigation in the blind is task-dependent (Kupers et al., 2010; Gagnon et al., 2012), because hippocampus is more likely recruited during the initial formation of the cognitive map and not during its retrieval (Iaria et al., 2003).

The anterior hippocampus also had increased rsFCs with brain regions related to spatial processing in the blind, which is consistent with a previous observation of increased volume in the anterior hippocampus in the blind (Fortin et al., 2008; Leporé et al., 2009). The “place cells” are also found in the anterior part of the hippocampus (Maurer et al., 2005; Kjelstrup et al., 2008). Thus, the structural and rsFC changes of the anterior hippocampus may reflect the enhanced use of non-visual spatial memory to compensate for the visual deprivation-induced deficit. Another possibility is that the increased rsFC of the anterior hippocampal parts may be also related to an enhanced use of non-spatial memory, because the anterior hippocampus is frequently reported as a memory processing center for objects (Köhler et al., 2002), and the anterior hippocampus is connected with the anterior temporal system that mainly supports object and verbal memory (Ranganath and Ritchey, 2012).

### The possible functional relevance of the strengthened rsFC of the hippocampus in the blind

A major limitation of this study is that we had neither collected navigational- or spatial-related behavioral variables, nor performed a navigational task to clarify how the strengthened hippocampal rsFC in the blind would affect spatial processing both at the behavioral and the functional levels. However, we think that it may be associated with spatial navigational functions in the blind based on the following evidence: First, brain regions showing enhanced rsFC with the hippocampus were located in navigational-related network (Committeri et al., 2004; Feierstein et al., 2006; Kupers et al., 2010; Kravitz et al., 2011). Second, the hippocampal tails showed increased rsFC with more extensive regions than the hippocampal heads and bodies in the blind; the hippocampal tails have also exhibited stronger navigational-related activation in the blind (Gagnon et al., 2012) and larger volume in Taxi drivers with extensive navigation experience (Maguire et al., 2000) than the hippocampal heads. Third, in the sighted subject, the posterior hippocampus prefers to connect with the posterior medial system to support visual spatial memory (Ranganath and Ritchey, 2012).

If the strengthened hippocampal rsFC of the blindness really indicate the reorganization of the spatial navigational network, another question is how would it affect the spatial processing at the behavioral level? Sighted subjects prefer to code spatial information in the form of survey-like (simultaneous) representations (Ruotolo et al., 2012), which facilitates integration of spatial information. However, blind subjects tend to code spatial information in the form of route-like (sequential) representations (Ruotolo et al., 2012); this form of processing requires more cognitive efforts (Thinus-Blanc and Gaunet, 1997). Thus, blind subjects need greater efforts and more exercises to develop comparable navigational/spatial skills as sighted subjects, suggesting that the enhanced hippocampal rsFC in the blind may reflect experience-dependent plasticity. Additionally, sighted subjects prefer to process spatial information using an external coordinate (allocentric) frame of reference because of the simultaneously perceptive properties of vision. In contrast, blind individuals tend to rely on more egocentric and experience-based representations (Röder et al., 2004, 2008; Collignon et al., 2009; Pasqualotto et al., 2013). Furthermore, it was reported that visual experience is critical for the development of the allocentric frame for multisensory action control (Röder et al., 2007; Pasqualotto et al., 2013). Thus, the CB may predominantly use egocentric frame while the LB may use both egocentric and allocentric frames for non-visual navigation (Röder et al., 2004, 2008; Collignon et al., 2009). As a result, the increased hippocampal rsFC in the blind may be associated with the altered preference of navigational processing strategies from allocentric to egocentric representation of space (Zaehle et al., 2007; Kravitz et al., 2011), which is also indirectly supported by the functionality of brain regions that exhibited an enhanced rsFC with the hippocampus: in sighted subject, the occipito-parietal areas mainly participant in egocentric spatial processing (Zaehle et al., 2007; Kravitz et al., 2011), and the PCC/RSC are core nodes involved in coordinating and translating between egocentric and allocentric reference frames (Kravitz et al., 2011; Knight and Hayman, 2014). However, this hypothesis should be directly confirmed in future by integrating behavioral and neuroimaging information.

However, we could not excluded the possibility that the increased rsFC of the hippocampus may also reflect an enhanced use of non-spatial memory. In this study, we also found strengthened rsFC in the hippocampal head and body, and the brain regions showing increased rsFC with the head and body were highly overlapped with that with the tail. Furthermore, the anterior hippocampus is frequently reported as a memory processing center for objects (Köhler et al., 2002), and the anterior hippocampus is connected with the anterior temporal system that mainly supports object and verbal memory (Ranganath and Ritchey, 2012). It is critically important to collect spatial and non-spatial behavioral variables in the future to clarify this issue.

### Similar reorganization of the hippocampal intrinsic functional network in the CB and the LB

We initially hypothesized that the CB would exhibit stronger functional reorganization in the hippocampus than the LB because the brain has a stronger plastic potentials in response to visual deprivation within the critical period of development than thereafter. This hypothesis is supported by a series of studies on task-evoked activation (Voss et al., 2008; Collignon et al., 2009), glucose metabolism (Veraart et al., 1990), and connectivity (Leporé et al., 2010) in the occipital cortex in the blind. It is also supported by studies showing that the PHC had increased nodal importance in the anatomical network (Li et al., 2013) and PHC-hippocampus had increased functional connectivity density (Qin et al., 2015) in the CB than in the LB. However, we found both the CB and the LB demonstrated comparable strengthened hippocampal rsFC, which is consistent with previous studies reporting that both the CB and LB groups showed superior non-visual navigational skills (Fortin et al., 2008) and auditory motion perception (Lewald, 2013) compared to the sighted group; and the two blind groups did not differ in hippocampal volume (Fortin et al., 2008). In contrast to the consistent association between occipital plasticity and blindness onset ages, the inconsistent findings between hippocampal plasticity and blindness onset ages may be related to the differences in sensory inputs between the hippocampus and occipital cortex. The early visual areas predominantly receive visual inputs; however, the hippocampus can process spatial information from multiple sensory sources (Tamura et al., 1992; Moita et al., 2003; Pereira et al., 2007). Animal studies showing that the activity of hippocampal place cells in early visual-deprived rats is similar to that in sighted rats (Save et al., 1998). The lack of visual input alone at an early developmental stage would not dramatically influence the maturation of hippocampus because it can maturate by receiving inputs from non-visual sensory modalities. Thus, the enhanced hippocampal rsFC in both the CB and LB may be a reflection of experience-dependent plasticity because they would make greater efforts and more exercises to develop navigational/spatial skills than sighted subjects.

It should be noted that the low spatial resolution data is a limitation of this study in consideration of the small structure of hippocampus. For the restrictions by hardware equipment (Siemens Trio Tim 3.0-Tesla MR scanner with maximum gradient strength of 45 mT/m) and routine single-shot EPI sequence, it is difficult to satisfy both high spatial resolution acquisition and whole brain coverage within TR of 2 s. In present study, we focused on the functional connectivity between the hippocampal subregions and whole brain, so we adopted a relative lower resolution to reach a whole-brain coverage. Further studies using MRI scanner with higher-level hardware equipment (for example, a maximum gradient strength of 80 mT/m and 32-channels or more head coils) and simultaneous multi-slice acquisition technique may be preferable to obtain higher spatial resolution fMRI images of hippocampus.

## Conclusions

In summary, we found increased intrinsic functional coupling between the hippocampus (especially the hippocampal tail) and several navigational-related areas after visual deprivation, which may reflect enhanced loading of the hippocampal-related networks for non-visual memory processing. We also found the changes of hippocampal rsFC were similar between the CB and the LB, suggesting an experience-dependent rather than a developmental-dependent plasticity of the hippocampal intrinsic functional network.

## Author contributions

CY and TJ designed and supervised this study, WQ and YL performed the experiment. GM, DY, YL, and WQ analyzed the data. GM and DY drafted the manuscript. CY and WQ revised the manuscript.

### Conflict of interest statement

The authors declare that the research was conducted in the absence of any commercial or financial relationships that could be construed as a potential conflict of interest.
