# Supplementary material for: Enhanced Functional Coupling of Hippocampal Sub-regions in Congenitally and Late Blind Subjects
Source: Front Neurosci. 2017 Jan 10;10:612. doi: 10.3389/fnins.2016.00612 (PMC5222804; doi:10.3389/fnins.2016.00612)
Supplement: Supplementary file 1 [file DataSheet1.DOCX]

**Supplementary materials**

**Part 1 Tables**

Table S1: The detailed demographic information of blind subjects.

| Group | Gender | Age | Onset | Duration | Diagnosis |
| --- | --- | --- | --- | --- | --- |
| CB01 | M | 28 | 0 | 28 | Retinal dysplasia |
| CB02 | F | 28 | 0 | 28 | Retinal pigmentosa |
| CB03 | F | 27 | 0 | 15 | Optic atrophy |
| CB04 | M | 23 | 0 | 27 | Fundus illness |
| CB05 | M | 24 | 0 | 4 | Eye dysplasia |
| CB06 | F | 27 | 0 | 17 | Retinal pigmentosa |
| CB07 | F | 20 | 0 | 19 | Unknown |
| CB08 | M | 22 | 0 | 6 | Retinal dystrophia |
| CB09 | M | 30 | 0 | 15 | Congenital cataract, Eyeball dystrophia |
| CB10 | M | 22 | 0 | 23 | Congenital cataract |
| CB11 | M | 27 | 0 | 24 | Optic atrophy |
| CB12 | F | 20 | 0 | 10 | Microphthalmus |
| CB13 | M | 23 | 0 | 20 | Unknown |
| CB14 | M | 39 | 0 | 27 | Pupil hypoplasia, Microphthalmus |
| CB15 | M | 36 | 0 | 7 | Fundus hypoplasia |
| CB16 | M | 29 | 0 | 13 | Unknown |
| CB17 | F | 21 | 0 | 12 | Fundus hypoplasia |
| CB18 | M | 31 | 0 | 4 | Microphthalmus |
| CB19 | F | 27 | 0 | 13 | Congenital cataract |
| CB20 | M | 28 | 0 | 23 | Fundus hypoplasia |
| LB01 | M | 35 | 20 | 8 | Detached retina |
| LB02 | M | 26 | 22 | 4 | Congenital cataract, Iris and fundus defect |
| LB03 | M | 39 | 22 | 20 | Microphthalmus |
| LB04 | M | 33 | 14 | 22 | Detached retina |
| LB05 | M | 24 | 18 | 11 | Congenital amblyopia |
| LB06 | M | 28 | 13 | 12 | Detached retina |
| LB07 | M | 27 | 17 | 16 | Glaucoma |
| LB08 | M | 35 | 15 | 10 | Detached retina |
| LB09 | M | 39 | 32 | 30 | Retinal pigmentosa |
| LB10 | M | 27 | 14 | 11 | Congenital glaucoma |
| LB11 | M | 28 | 16 | 16 | Glaucoma, Cataract |
| LB12 | M | 25 | 21 | 22 | Optic dystrophia |
| LB13 | M | 30 | 17 | 5 | Detached retina |
| LB14 | M | 43 | 20 | 27 | Detached retina |
| LB15 | F | 25 | 17 | 20 | Microphthalmus |
| LB16 | M | 29 | 25 | 13 | Ocular hypertension |
| LB17 | F | 24 | 13 | 4 | Fundus haemorrhage |
| LB18 | F | 28 | 16 | 5 | Iridocyclitis |
| LB19 | M | 31 | 15 | 26 | Detached retina, Vitreous opacities |
| LB20 | F | 30 | 20 | 23 | Retinal pigmentosa, Optic atrophy |
| LB21 | M | 26 | 15 | 39 | Detached retina, Congenital cataract |
| LB22 | M | 29 | 13 | 7 | Glaucoma |
| LB23 | M | 33 | 28 | 36 | Retinal pigmentosa |
| LB24 | M | 27 | 14 | 29 | Detached retina |
| LB25 | F | 21 | 17 | 21 | Fundus inflammation |
| LB26 | M | 23 | 18 | 8 | Optic atrophy |
| LB27 | M | 41 | 15 | 13 | Glaucoma |
| LB28 | M | 41 | 34 | 7 | Optic atrophy |
| LB29 | F | 28 | 20 | 7 | Congenital cataract |
| LB30 | M | 30 | 17 | 14 | Detached retina |
| LB31 | F | 23 | 16 | 31 | Congenital cataract |
| LB32 | M | 31 | 24 | 12 | Congenital cataract |
| LB33 | F | 31 | 17 | 7 | Optic atrophy |
| LB34 | F | 27 | 15 | 8 | Unknown |
| LB35 | F | 20 | 13 | 9 | Unknown |
| LB36 | M | 30 | 22 | 11 | Retinopathy |
| LB37 | F | 41 | 32 | 27 | Retinal pigmentosa |
| LB38 | M | 38 | 27 | 9 | Optic atrophy |
| LB39 | F | 34 | 25 | 5 | Fundus hemorrhage |
| LB40 | F | 25 | 20 | 28 | Glaucoma |
| LB41 | M | 34 | 17 | 17 | Glaucoma |
| LB42 | M | 31 | 18 | 13 | Optic neuritis |

Table S2. Correlations between hippocampal rsFC and demographic variables

| Hippocampal sub-regions | Target regions | Duration of CB | | Duration of LB | | Onset age of LB | |
| --- | --- | --- | --- | --- | --- | --- | --- |
|  |  | r | p | r | p | r | p |
| Left head | PCC | 0.17 | 0.50 | -0.03 | 0.86 | -0.03 | 0.87 |
| Left tail | L_AG | -0.07 | 0.78 | 0.12 | 0.44 | 0.08 | 0.62 |
| Left tail | L_MOT | -0.09 | 0.70 | 0.18 | 0.26 | -0.09 | 0.58 |
| Left tail | PCC | -0.11 | 0.65 | 0.10 | 0.54 | 0.11 | 0.50 |
| Left tail | R_AG | 0.15 | 0.53 | 0.13 | 0.42 | -0.01 | 0.96 |
| Left tail | R_ITG | -0.02 | 0.93 | 0.08 | 0.63 | 0.12 | 0.47 |
| Left tail | R_MFG | -0.24 | 0.33 | 0.12 | 0.46 | -0.07 | 0.65 |
| Left tail | R_OFC | -0.35 | 0.14 | 0.11 | 0.50 | -0.22 | 0.17 |
| Left tail | R_MOT | 0.03 | 0.90 | 0.13 | 0.43 | 0.00 | 0.99 |
| Right head | R_ITG | 0.08 | 0.75 | 0.10 | 0.55 | 0.08 | 0.61 |
| Right head | R_MOT | 0.01 | 0.96 | 0.09 | 0.56 | 0.09 | 0.58 |
| Right body | R_MOT | -0.07 | 0.78 | 0.16 | 0.32 | 0.01 | 0.97 |
| Right tail | L_AG | 0.03 | 0.90 | 0.03 | 0.87 | 0.11 | 0.51 |
| Right tail | L_MOT | 0.00 | 0.99 | 0.04 | 0.81 | 0.03 | 0.83 |
| Right tail | PCC | -0.11 | 0.64 | -0.05 | 0.74 | 0.17 | 0.28 |
| Right tail | R_aITG | -0.10 | 0.69 | 0.04 | 0.80 | -0.12 | 0.44 |
| Right tail | R_MOT | 0.08 | 0.75 | 0.10 | 0.52 | 0.08 | 0.62 |
| Right tail | R_pITG | -0.24 | 0.32 | 0.02 | 0.90 | 0.11 | 0.48 |

Note: Partial correlation analyses controlling for gender effect (P <0.05, uncorrected). AG = [angular gyrus](http://en.wikipedia.org/wiki/Angular_gyrus), aITG = anterior inferiortemporal gyrus, CB= congenitally blind, ITG = inferiortemporal gyrus, LB=late blind, MFG = middle frontal gyrus, MOT = middle [occipito-temporal](http://en.wikipedia.org/wiki/Middle_temporal_gyrus) conjunction, OFC = orbital frontal cortex, PCC = [posterior cingulate cortex](http://en.wikipedia.org/wiki/Posterior_cingulate_cortex), pITG = posterior inferiortemporal gyrus.

**Part 2 Figures**


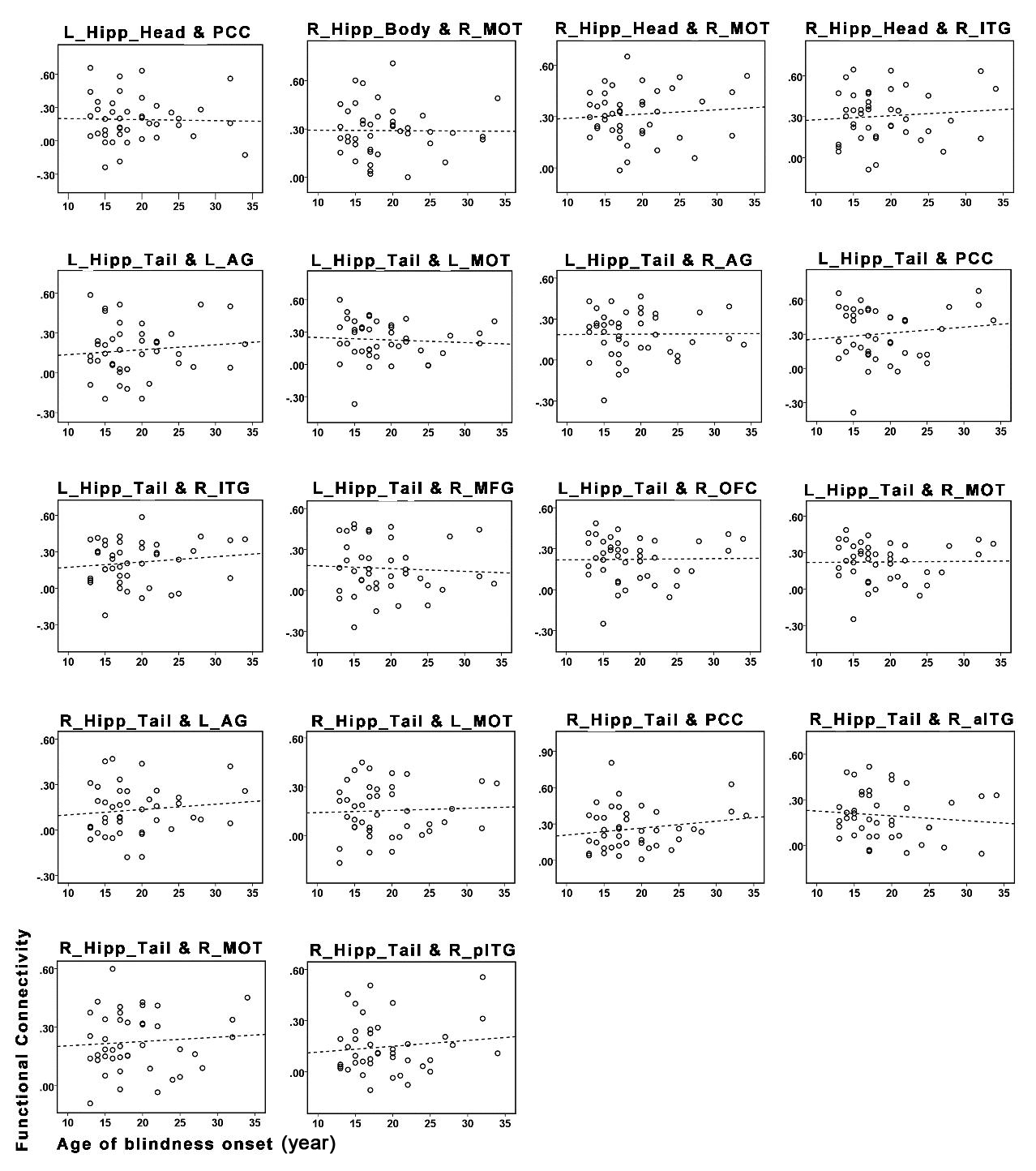


Figure S1. Correlations between the hippocampal rsFC and age of blindness onset in the LB. No significant correlation is found. AG = [angular gyrus](http://en.wikipedia.org/wiki/Angular_gyrus), aITG = anterior inferiortemporal gyrus, Hipp_Body = hippocampal body, Hipp_Head = hippocampal head, Hipp_Tail = hippocampal tail, ITG = inferiortemporal gyrus, MFG = middle frontal gyrus, MOT = middle [occipito-temporal](http://en.wikipedia.org/wiki/Middle_temporal_gyrus) conjunction, OFC = orbital frontal cortex, PCC = [posterior cingulate cortex](http://en.wikipedia.org/wiki/Posterior_cingulate_cortex), pITG = posterior inferiortemporal gyrus.


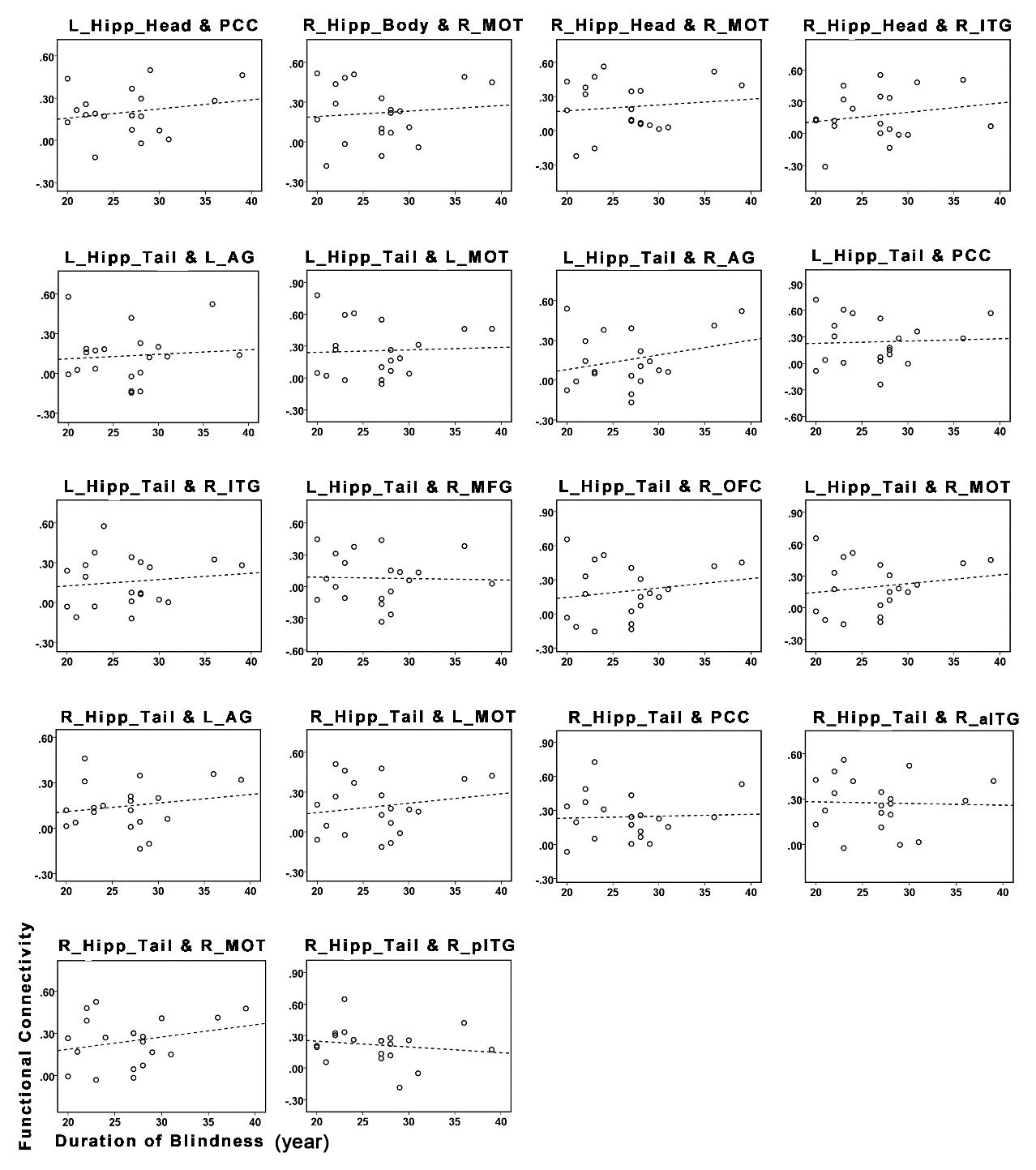


Figure S2. Correlations between the hippocampal rsFC and duration of blindness in the CB. No significant correlation is found. AG = [angular gyrus](http://en.wikipedia.org/wiki/Angular_gyrus), aITG = anterior inferiortemporal gyrus, Hipp_Body = hippocampal body, Hipp_Head = hippocampal head, Hipp_Tail = hippocampal tail, ITG = inferiortemporal gyrus, MFG = middle frontal gyrus, MOT = middle [occipito-temporal](http://en.wikipedia.org/wiki/Middle_temporal_gyrus) conjunction, OFC = orbital frontal cortex, PCC = [posterior cingulate cortex](http://en.wikipedia.org/wiki/Posterior_cingulate_cortex), pITG = posterior inferiortemporal gyrus.


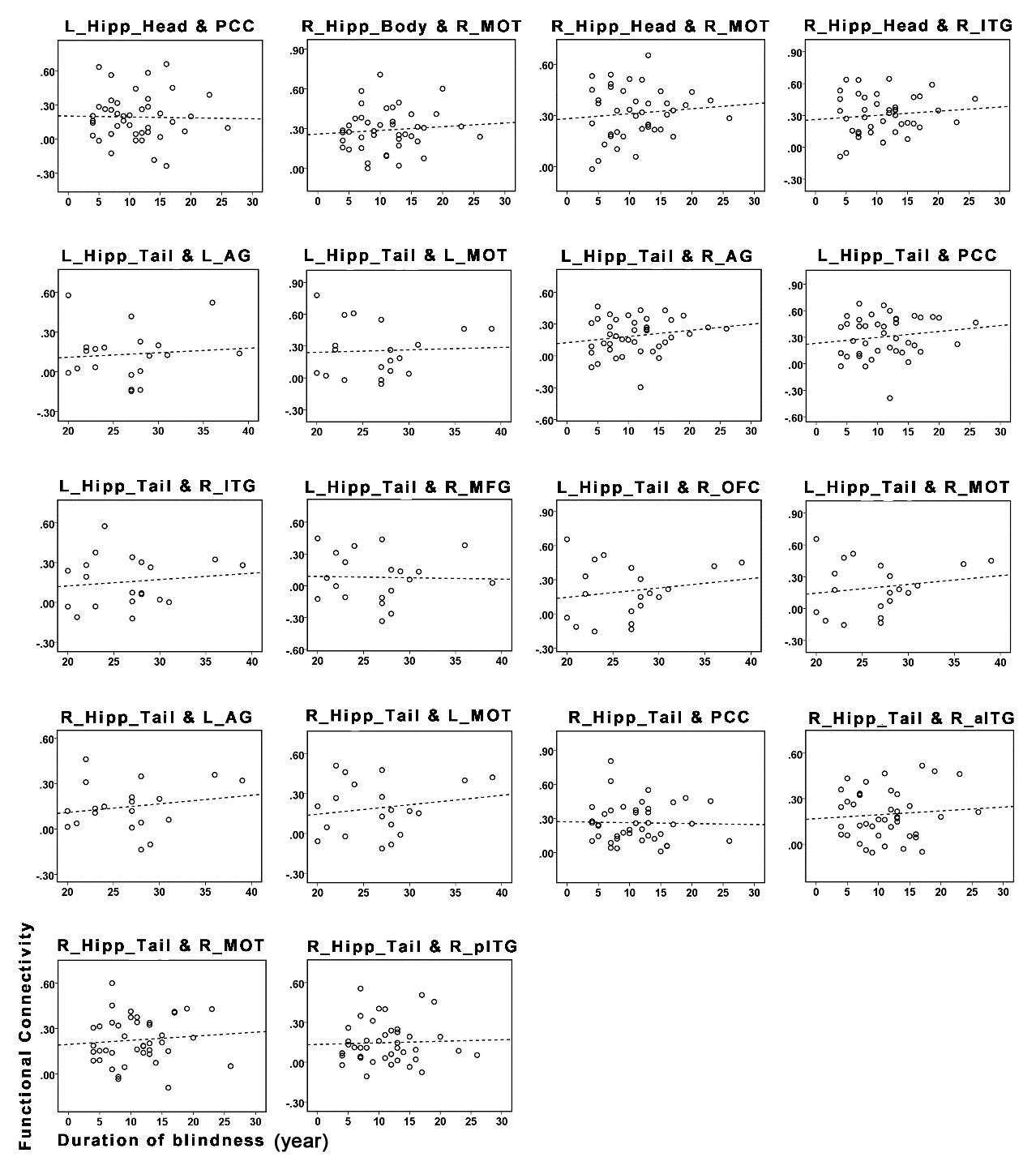


Figure S3. Correlations between the hippocampal rsFC and duration of blindness in the LB. No significant correlation is found. AG = [angular gyrus](http://en.wikipedia.org/wiki/Angular_gyrus), aITG = anterior inferiortemporal gyrus, Hipp_Body = hippocampal body, Hipp_Head = hippocampal head, Hipp_Tail = hippocampal tail, ITG = inferiortemporal gyrus, MFG = middle frontal gyrus, MOT = middle [occipito-temporal](http://en.wikipedia.org/wiki/Middle_temporal_gyrus) conjunction, OFC = orbital frontal cortex, PCC = [posterior cingulate cortex](http://en.wikipedia.org/wiki/Posterior_cingulate_cortex), pITG = posterior inferiortemporal gyrus.
